# Evaluation of the Phenolic Composition and Biological Activities of Six Aqueous Date (*Phoenix dactylifera* L.) Seed Extracts Originating from Different Countries: A Comparative Analysis

**DOI:** 10.3390/foods13010126

**Published:** 2023-12-29

**Authors:** Aseel Swaidan, Bilal Azakir, Susanne Neugart, Naim Kattour, Elie Salem Sokhn, Tareq M. Osaili, Nada El Darra

**Affiliations:** 1 Department of Nutrition and Dietetics, Faculty of Health Sciences, Beirut Arab University, Tarik El Jedidah, Riad El Solh, P.O. Box 115020, Beirut 1107 2809, Lebanon; aseelswaidan00@gmail.com; 2 Molecular and Translational Medicine Laboratory, Faculty of Medicine, Beirut Arab University, Beirut 1107 2809, Lebanon; b.azakir@bau.edu.lb; 3 Division of Quality and Sensory of Plant Products, Department of Crop Sciences, Georg-August-Universität Göttingen, 37073 Göttingen, Germany; 4 Department of Biology, Faculty of Arts and Sciences, University of Balamand, P.O. Box 100, Tripoli 1100 2807, Lebanon; 5 Department of Medical Laboratory Technology, Faculty of Health Sciences, Beirut Arab University, Beirut 1107 2809, Lebanon; e.sokhn@bau.edu.lb; 6 Department of Clinical Nutrition and Dietetics, College of Health Sciences, University of Sharjah, Sharjah P.O. Box 27272, United Arab Emirates

**Keywords:** date seed extract, polyphenols, antioxidant, antibacterial, anticancer, date seed oil

## Abstract

Date seeds, which are the main by-products of date fruit consumption, were shown to possess promising biological activities and health benefits with minimal human use. The present investigation analyzed and compared the phenolic content of six date seed varieties from four different origins (Khudari, Sakai, and Safawi from Saudi Arabia, Majdool from Jordan, Zahdi from Iraq, and Kabkab from Iran). The aqueous extracts were examined for possible antioxidant, antibacterial, and anti-tumor potential. Date seed oil was extracted, and fatty acid profiles were compared. The results revealed that date seeds are a rich source of polyphenols, which have been linked to biological activities. Furthermore, the phenolic content seemed highly dependent on the variety, where Kabkab had the highest TPC value (271.2 mg GAE/g DM) while Majdool had the lowest value (63.2 mg GAE/g DM). Antioxidant activities of all varieties were highly correlated with the total phenolic content. The antibacterial investigation demonstrated that the Sakai variety possessed the dominant activity, whereas Majdool showed no activity. The results further indicated the sensitivity of both *Staphylococcus aureus* and *Bacillus cereus*, with a stronger effect against *B. cereus*, while no effect was observed against Gram-negative strains (*Salmonella* Typhi and *Escherichia coli*). All varieties were able to decrease colon and lung cancer cell viability, especially Khudari and Sakai, with stronger effects against colon cancer cells. Analysis of date seed oil showed high oleic acid content, especially in Sakai. The findings suggest that date seeds are promising candidates for future pharmaceutical applications as nutraceuticals to help combat certain illnesses, as well as functional foods and natural additives that boost the nutritional value of food products, increase their shelf lives, and improve the overall health of consumers.

## 1. Introduction

For over 6000 years, date palm fruit (*Phoenix dactylifera* L.) have remained one of the most popular staple fruits to be cultivated. Dates are grown and harvested in various countries around the world, with Egypt, Saudi Arabia, Iran, and Iraq being among the top four [1]. According to data from the Food and Agriculture Organization (FAO), Egypt was reported to be the world’s top producer of dates in 2021, followed by Saudi Arabia and Iran, with total worldwide production reaching around 9.66 million metric tons [2].

Date seeds, a major by-product of date fruit consumption, usually weigh between 10% and 15% of the total weight of the date fruit [3]. The widespread consumption of date palm fruit raises concerns about the disposal of its by-products, mainly seeds, and the potential adverse consequences of their unsafe disposal. In fact, the available literature on the disposal of date seeds lacks sufficient information. However, some studies have indicated that they are usually discarded or used as animal feed for livestock such as cattle, sheep, and poultry [4,5].

Interestingly, numerous studies evaluated the high bioactive molecules present in date seeds, particularly their phenolic content and composition, as they were found to be rich in phenolic compounds including flavonoids (such as hesperidin, quercetin, and kaempferol), phenolic acids (caffeic acid, epicatechin, catechol, and chlorogenic acid), as well as other compounds such as carotenoids and dietary fibers (mainly pectin, β-glucan, and arabinoxylan) [6,7]. It was shown that the country of origin and variety appeared to have a significant impact on the composition of date seeds. For instance, a study found that the phenolic content among 18 different varieties averaged 3411.8 ± 717.4 mg GAE/100 g, with a wide significant variation between the examined varieties [8]. The overall phenolic content of date seeds was reported to be higher than that of edible flesh [7].

Date seeds were shown to exhibit a powerful ability to quench reactive oxygen species (ROS) and protect cellular components like DNA from free radicals, which renders them a potential candidate for assisting in the prevention and management of chronic illnesses such as diabetes, hypertension, inflammatory conditions, and even disrupted lipid profiles [9]. Date seeds also possess antibacterial properties, which are of particular interest, as researchers are concerned about the recent growth in drug-resistant microorganisms. It would be crucial to evaluate the antimicrobial activity of such by-products. Depending on how effective this impact is, date seeds may be able to replace or even act as a synergy to help reduce the dosage of the antibiotic prescription. A substantial association was discovered between the amount of polyphenols present in date seeds and their antibacterial properties [10]. Date seeds have been shown to considerably reduce nucleic acid production in both Gram-positive and Gram-negative bacterial strains; however, greater efficacy was dominant against Gram-positive strains, particularly *Staphylococcus aureus* [11]. Along with these auspicious properties, date seeds have demonstrated promising anti-tumor activity against various cancer cell lines, including human breast adenocarcinoma (MCF-7 and MDA-MB-231), human colon adenocarcinoma (Caco-2), human hepatocyte carcinoma (HepG2), human prostate adenocarcinoma (PC-3), and pancreatic cancer (Mia-Pa-Ca-2), at variable concentrations with time-dependent responses [12,13,14].

In addition to their excellent phenolic composition, date seeds contain considerable amounts of fat, ranging from 7% to 13% [15]. Date seed oil is composed of oleic, linoleic, lauric, palmitoleic, and linoleic acids, as well as other high-quality saturated and unsaturated fatty acids, with oleic acid predominating and ranging between 41 and 58.8% [16].

In light of their bioactive compounds, including polyphenols, which were previously reported to possess interesting biological activities and health benefits [17,18,19,20], the valorization of date seeds’ polyphenols could offer a possible natural strategy to help combat many chronic conditions and foodborne illnesses. Furthermore, they can be employed in food industries as a natural antioxidant source added to food products, providing an alternative to synthetic ones such as butylated hydroxyanisole (BHA) and butylated hydroxytoluene (BHT). Accordingly, the current study aimed to evaluate and compare the total phenolic content and phenolic profile of aqueous date seed extracts of six different varieties (Khudari, Sakai, Safawi, Majdool, Zahdi, and Kabkab) originating from four different countries (Saudi Arabia, Jordan, Iraq, and Iran). Furthermore, the study was conducted to investigate and compare various biological activities of the date seed extracts, mainly antioxidant, antibacterial, and anti-tumor. In addition, date seed oils were extracted, and their fatty acid composition was evaluated. To our knowledge, so far, no prior research was conducted to examine three biological activities (antioxidant, antibacterial, and anti-tumor) in the same study, for such a diverse range of date seed aqueous extracts (six varieties). Moreover, date seeds have not been previously studied in Lebanon. We believe that the findings of this study will identify the optimum variety of date seeds that possess the strongest biological activity and suggest date seeds as a potential promising candidate for use in the fields of pharmaceutical and/or food production to help prevent diseases and boost the nutritional value of food products.

## 2. Materials and Methods

### 2.1. Chemicals and Reagents

All chemicals and reagents used in this study were of analytical grade, and the water was distilled. Folin–Ciocalteu reagent, sodium carbonate anhydre (NaCO₃), gallic acid, ABTS^●+^ (2,2′-azino-bis (3-ethylbenzothiazoline-6-sulfonic acid) diammonium salt) (≥98%), isopropanol, diethyl ether, and tert-butyl methyl ether, fetal bovine serum (FBS) (cat. no. F9665), Dulbecco’s Modified Eagle Medium/Nutrient Mixture F-12 (DMEM) (cat. no. D8437), Roswell Park Memorial Institute (RPMI) 1640 (cat. no. R2405), phosphate-buffered saline (PBS) (cat. no. D8537T), trypsin (cat. no. T3924), trypan blue (cat. no. T8154), MTT reagent (3-(4,5-dimethylthiazol-2-yl)-2,5-diphenyl-2H-tetrazolium bromide) (cat. no. M5655) were purchased from Sigma-Aldrich; Merck KGaA, F. Penicillin-streptomycin (cat. no. L0022) was purchased from Biowest, Nuaillé, France. Mueller Hinton Agar (MHA), Mueller Hinton Broth (MHB), and MacConkey agar (MAC) were obtained from HIMEDIA (Mumbai, India). The radical DPPH (2,2-diphenyl-1-picrylhydrazyl) (95%) and Trolox^®^ (97%) were purchased from Thermo Fisher (Kandel, Germany). Acetonitrile (ACN; HPLC grade), methanol (MeOH; HPLC grade), potassium persulfate (≥99%), and HPLC standards were purchased from Carl Roth (Karlsruhe, Germany).

### 2.2. Date Seed Sample Preparation

This study included six different varieties of date seeds: three from Saudi Arabia (Khudari, Sakai, and Safawi), one from Jordan (Majdool), one from Iraq (Zahdi), and one from Iran (Kabkab). One kilogram of each variety was purchased from a well-known date store in Lebanon, where dates were randomly chosen without any preference in appearance, color, or size. Information regarding the origin of each variety was provided by the supplier. The date fruits were pitted to obtain their seeds and the latter were rinsed with potable water to remove any sticky flesh then dried overnight at room temperature. After that, using a hammer, date seeds were crushed into small particles before being ground into fine powder with the aid of an electric grinder (Moulinex 255, Paris, France). The powdered samples were allowed to pass through a 0.5 mm mesh before being frozen for subsequent use.

### 2.3. Solid–Liquid Extraction (SLE)

For SLE, date seed powder was mixed with distilled water in a ratio equivalent to 1:100 (*w*/*v*). The solution was stirred continuously at room temperature using a magnetic stirrer. Multiple extraction was carried out, with each extraction lasting 20 min and repeated three times (for a total duration of one hour). After every single extraction, the extract was filtered with a glass wool, and the solid residue was collected and re-extracted with another 100 mL of distilled water. The obtained crude extracts were then centrifuged at 6000 rpm for 10 min.

### 2.4. Lyophilization of Date Seed Extracts

For lyophilization of our samples, each date seed’s water extract was separated into four separate flasks and frozen for twenty-four hours at a temperature of −20 °C. For a period of 48 h, the frozen flasks were attached directly to the ports of a manifold benchtop lyophilizer (TELSTAR^®^ CRYODOS—50, Madrid, Spain). All powder extracts were collected and kept at −20 °C in screw cap vials until employed in subsequent experiments.

### 2.5. Determination of Total Phenolic Content (TPC)

The total phenolic content of date seeds was quantified according to the Folin–Ciocalteu method [21] and expressed as mg gallic acid equivalent per gram of sample dry matter (mg GAE/g DM) using the equation obtained from the gallic acid calibration curve (R^2^ > 0.99). In brief, 0.1 mL of the sample was mixed with 0.5 mL of Folin–Ciocalteu solution. After 6 min, 0.4 mL of sodium carbonate solution was added and vortexed. The blank was prepared using the same process but with 0.1 mL of distilled water instead of the sample. Tubes were covered with aluminum foil and placed in a water bath at 60 °C for 10 min before being placed in a refrigerator at 4 °C for a further 10 min. Finally, the absorbance was measured at 750 nm using a UV-Vis spectrophotometer (OPTIMA SP-300, Kanagawa, Japan).

### 2.6. Determination of Phenolic Compounds in Date Seed Extracts

#### 2.6.1. Sample Preparation

The chromatographic analysis was carried out at Georg-August-Universität Göttingen in Germany. The extract was prepared according to a method described by Neugart et al. [22]. In brief, 20 mg of the lyophilized samples were extracted for 1 h at room temperature with 600 μL of 60% aqueous methanol under continuous stirring. The extracts were then centrifuged at 19,000× *g* for 10 min before the supernatants were collected. The extraction was performed twice more using 400 μL and 200 μL of 60% aqueous methanol for 20 min and 10 min, respectively. The centrifugation step was followed by each extraction and the collected supernatants were dried using a rotary evaporator. Subsequently, the dried residues were dissolved in 200 μL distilled water, and the extracts were filtered through centrifuge tube filters with a 0.22 μm cellulose acetate membrane (Corning^®^Costar^®^ Spin-X^®^, Sigma Aldrich Chemical Co., St. Louis, MO, USA) for HPLC analysis.

#### 2.6.2. High-Performance Liquid Chromatography (HPLC-DAD-ESI-MS) Analysis

The identification and quantification of the phenolic compounds in date seed extracts were carried out according to a method described by Engelhardt et al. [23], using a high-performance liquid chromatography (HPLC) system (Maryland, Columbia, Shimadzu Prominence 20) equipped with a refrigerated SIL-20AC HT autosampler, an LC-20 AT liquid chromatograph quaternary pump, a CTO-10AS VP column oven, a DGU-20A5 degasser, an SPD-M20A diode array detector (DAD) (detection wavelength: 280 nm for proanthocyanidins and 320 nm for caffeic acid derivatives), and a CBM-20A communication bus module. Furthermore, a Supelco Ascentis^®^ Express F5150 × 3.0 mm, 5 µm was utilized, along with a Supelco Guard column 5.0 × 3.0 mm, 5 µm, and a 0.2-micron SST Frits for Ultra Line. The column temperature was set to 30 °C. The mobile phase consisted of Eluent A (1% acetic acid) and Eluent B (100% acetonitrile). The separation was achieved using the following gradient: 0–7 min, 5% B; 7–30 min, 5–20% B; 30–49.5 min, 20–90% B; 49.5–52 min 90% B, 52–52.7 min 90–5% B; 52.7–59 min 5% B. The flow rate was 0.3 mL/minute, and the injection volume was 30 µL. Standard calibration curves for phenolic compounds (Procyanidin B2 (PhytoLab, Vestenbergsgreuth, Germany) and chlorogenic acid for caffeic acid derivatives (Carl Roth, Germany)) were used for quantification. The limit of detection (LOD; factor 3.3) and quantification (LOQ; factor 10) were obtained from the calculated standard error of the intercepts and the slopes of the calibration curves. Finally, for the identification of phenolic compounds, samples were measured in a negative ion mode using an Agilent 6460 HPLC-QQQ equipped with electrospray ionization (ESI) interface, applied to perform mass spectrometry (MS) at the Division of Molecular Phytopathology and Mycotoxin Research of the Georg-August-Universität Göttingen, Germany. Ion source parameters were set as follows: capillary voltage = +4000/−4000 V, gas temperature = 350 °C, gas flow rate = 13 L/minute, and nebulizer = 60 psi. Fragmentation voltage (135 V) was applied for collision-induced dissociation.

### 2.7. Antioxidant Activity of Date Seed Extracts

The antioxidant properties of the six date seed cultivars were evaluated at Georg-August-Universität Göttingen, Germany, by applying two commonly used in vitro antioxidant assays as described below.

#### 2.7.1. ABTS Assay

The ABTS^•+^ radical cation decolorization method was used to measure the free radical scavenging activity of date seed extracts [23]. In brief, the stock solution was made by mixing 9.6 mg of ABTS and 1.66 mg of potassium persulfate with 25 mL of distilled water. The solution was then incubated in the dark at room temperature for 12 to 16 h to allow complete radicalization. The working solution was prepared by diluting 5 mL of the stock solution with 100% methanol up to 25 mL. After that, 10 µL of the samples were mixed with 150 µL of the working solution and then incubated for 5 min. After 1 min of orbital shaking at medium speed, followed by a delay of 1 min, the absorbance was measured at 734 nm using a spectrophotometer against 100% methanol blank solution. The control was prepared using the same protocol but with 10 µL of distilled water instead of the sample. A standard curve ranging from 0.025 to 0.7 mM Trolox scavenging activity (R^2^ > 0.99) was prepared, and the results were expressed as Trolox equivalent antioxidant capacities (*TEAC*) [mmol TE/g sample (dry weight)] using the following equation:

TEAC=C×V×DF/m

where *C* is the concentration obtained from the Trolox calibration curve (mmoL/mL), *V* is the total volume of the sample prepared (mL), *DF* is the dilution factor, and *m* is the weight of the dry sample (g).

#### 2.7.2. DPPH Assay

The antioxidant activity of date seed extracts was also assessed by their ability to scavenge the ”stable” free radical 2,2-diphenyl-1-picrylhydrazyl• (DPPH•) [24]. The DPPH solution was prepared by dissolving 7.88 mg in 100 mL of 100% methanol and incubated in the dark at room temperature for 2 h. After that, 20 µL of the sample was mixed with 180 µL of the prepared DPPH solution and incubated in the dark at room temperature for 28 min. Orbital shaking was performed at medium speed for 1 min, and after 1 min of delay, the absorbance was measured at 515 nm using a spectrophotometer. The control was prepared with the same protocol but with 20 µL of distilled water instead of the sample; the blank consisted of 100% methanol. A standard curve ranging from 0.025 to 0.7 mM Trolox was prepared (R^2^ > 0.99).

In addition, %*DPPH inhibition* was calculated according to the following equation:

$$\begin{array}{c} \%DPPH\;inhibition\\ =\frac{Absorbance\;of\;control-Absorbance\;of\;sample}{Absorbance\;of\;control\;}\\ \times 100 \end{array}$$

### 2.8. Antibacterial Activity of Date Seed Extracts

#### 2.8.1. Sample Preparation

After conducting several trials of the disk diffusion experiment using different solid-solvent ratios (1:100, 1:5, and 1:2), the 1:2 ratio was chosen as the optimum. The total phenolic content of the extracts at this ratio was determined using the same method described previously [21]. For this experiment, the aqueous date seed extracts were sterilized by filtration using disposable 0.22 µm syringe filters.

#### 2.8.2. Inoculum Standardization

The antibacterial properties of the six date seed varieties were evaluated against two Gram-positive bacterial strains: *Staphylococcus aureus* (ATCC 25923) and *Bacillus cereus* (ATCC14579 ), as well as two Gram-negative bacterial strains: *Salmonella* Typhi (ATCC 14028) and *Escherichia coli* (ATCC 25922). Lebanese Agricultural Research Institute (LARI) provided these strains. The bacterial strains were subcultured on Mueller Hinton agar, except for *Salmonella* and *E. coli*, which were subcultured on MacConkey agar [25,26] and incubated overnight at 37 °C. The bacterial inoculum was prepared by mixing bacteria with a sterile saline solution (0.9%) and adjusting the turbidity so that it matched that of the prepared 0.5 McFarland (10^8^ CFU/mL).

#### 2.8.3. Antibacterial Screening: Disk Diffusion Method

Antibacterial screening was performed according to the disk diffusion method [27]. In brief, Mueller Hinton agar was prepared and poured into sterile Petri dishes then allowed to cool at room temperature. After that, the prepared bacterial suspensions (10^8^ CFU/mL) were evenly streaked on the agar surface using sterile cotton swabs. After 10 min, using heat sterilized forceps, sterile disks (5 mm) were positioned in each Petri dish. Next, 20 µL of the sample was placed on each disk. Distilled water was used as the negative control, while Gentamycin (50 μg) served as the positive control. The extracts were allowed to absorb by the agar for 15–20 min at room temperature prior to incubation at 37 °C. After 24 h, the diameters of the inhibition zones were measured and reported in millimeters (mm).

#### 2.8.4. Determination of Minimum Inhibitory Concentration (MIC)

The MIC was determined for the two varieties of date seeds that possessed the strongest antibacterial activities in the disk diffusion method (Khudari and Sakai).

In brief, six different concentrations of date seed extracts (83.3, 41.6, 20.8, 10.4, 5.2, and 2.6 mg/mL, starting with an initial concentration of 166.7 mg/mL) were prepared via the serial dilution method described as follows: In each sterile test tube, 1 mL of Mueller Hinton broth (MHB) was added. For the preparation of the first concentration (83.3 mg/mL), 1 mL of date seed extract of initial concentration (166.7 mg/mL) was added to MHB and mixed by vortex. The other concentrations were obtained by mixing 1 mL of the just-before concentration with MHB. After serial dilution, 1 mL from each of the four prepared bacterial suspensions was added to each tube to assess the ability of each extract to inhibit bacterial growth and determine the MIC.

### 2.9. Anti-Tumor Activity of Date Seed Extracts

The anti-tumor capacities of the different date seed varieties were investigated using MTT assay [28].

#### 2.9.1. Cell Lines

The anti-tumor activity of date seeds was examined against two cancer cell lines: the human colorectal cell line (HT-29) (cells were gifts from Wassim Abou El kheir)and the human lung cell line (A549) (cells were gifts from Salem Chouaib). HT-29 cell line was cultured in Roswell Park Memorial Institute (RPMI) 1640 medium, whereas A549 was cultured in Dulbecco’s Modified Eagles Medium (DMEM). Both media were supplemented with 10% fetal bovine serum (FBS), 2 mM L-glutamine, and 1% penicillin/streptomycin. Cells were incubated in a humidified atmosphere with 5% CO_2_ at 37 °C.

#### 2.9.2. Seeding and Treatment

For this experiment, 7 × 10^3^ cells per well were seeded in a 96-well plate in their corresponding culture medium and incubated for 24 h with 5% CO_2_ at 37 °C. After that, a stock solution (1 mg/mL) from each of the different date seed varieties was freshly prepared just before treating the cells by dissolving lyophilized date seed powder in dimethyl sulfoxide (DMSO) solution. From the stock solution, working solutions with different concentrations (10, 25, 50, 65, 75, and 100 μg/mL) were prepared. After 24 h, the seeded cells were treated with 100 μL of these different concentrations of date seed samples. The control wells contained cells in their appropriate media only, while mock wells consisted of cells in media with 0.2% DMSO.

#### 2.9.3. MTT Assay

Cell viability was evaluated using an MTT assay. Medium was removed from the wells and replaced with 50 μL of a 5 mg/mL MTT solution. The blank consisted of media with 10% MTT without cells. After 4 h of incubation, 50 μL of DMSO was added into the wells, and plates were gently shaken for 2 min to dissolve the formed formazan crystals. The absorbance was measured at 620 nm using an ELISA reader (Thermo Fisher Scientific, Boston, MA, USA). The experiment was repeated in triplicate. Results were reported in terms of % cell viability ± SD using the following equation:

$$\%\;Cell\;viability=\frac{\left(At-Ab\right)}{Am}\times 100$$

where *At* is the absorbance of the tested sample, *Ab* is the blank absorbance, and *Am* is the absorbance of the mock.

Finally, IC_50_ (the concentration that reduced cell viability to 50%) was determined for each variety against each cell line using microcal origin software by non-linear regression analysis using the dose–response equation. IC_50_ was used to compare the cytotoxic activities of the tested date seed varieties.

### 2.10. Extraction of Date Seed Oil

Soxhlet extraction method was used to extract oil from date seeds [29]. In brief, 8 g of date seed powder was extracted by 150 mL diethyl ether. The temperature was set at 60–70 °C. The extraction was stopped after 6 h, and oil was separated from the mixture using a rotary evaporator under vacuum at 40 °C with a rotation speed of 30 rpm.

### 2.11. Determination of Fatty Acid Compounds in Date Seed Oil

The fatty acid profile of the extracted date seed oil was analyzed at Georg-August-Universität Göttingen, Germany, using a gas chromatography system equipped with mass spectrometry (GC/MS-QP2010 Ultra). For the formation of fatty acid methyl esters, the sample was mixed with tert-butyl methyl ether (1:1). The injection volume was 5 µL in split mode (split ratio 1:10). The quantitative analysis was conducted with a GC-2010 Plus (Shimadzu Deutschland GmbH, Duisburg, Germany) equipped with a flame ionization detector (FID). The FID temperature was set to 250 °C. Helium was used as the carrier gas with a column flow rate of 1.24 mL/minute. The temperature was adjusted to 35 °C (hold for 5 min) and then increased to 210 °C (5 °C/min). The final temperature was held for 20 min. To achieve compound separation, an SH-Stabilwax, 0.25 mm ID × 30 m length × 0.25 µm film thickness was selected. Compounds were confirmed by comparison with analytical standards and quantified using their corresponding peak areas.

### 2.12. Statistical Analysis

The results were reported as mean ± standard deviation. Statistical analysis was carried out using SPSS for Windows (version 20). To determine significance, data was examined using a one-way analysis of variance (ANOVA), followed by Tukey’s test for multiple comparisons. When *p*-value < 0.05, the results were considered significantly significant.

## 3. Results and Discussion

### 3.1. Total Phenolic Content (TPC) of Date Seeds

The total phenol content (TPC) of the various date seed cultivars is reported in Table 1 in terms of mg GAE/g sample DM. As can be noticed, TPC varied significantly across the six evaluated date seed varieties. On average, TPC was estimated to be 154.2 ± 1.3 mg GAE/g DM, ranging between 63.2 ± 0.1 and 271.2 ± 3.7 mg GAE/g DM. With respect to the order among varieties, the trend was as follows: Kabkab appeared to have the highest TPC value, followed by Zahdi, Sakai, Safawi, and Khudari, whereas Majdool had the lowest. It should be noted that the findings obtained cannot be directly compared to the available literature, as some of the tested varieties were not included in previous studies. It was noticed that the TPC varied significantly, even when comparing different studies that examined the same variety. This can be due to the fact that most prior research used solvents other than distilled water to extract polyphenols from date seeds. Furthermore, like with many other plants and fruit seeds, the total phenolic content and overall composition can vary due to differences in variety and origin. In addition, several factors, including climatic conditions, maturity, geographic location, irrigation, harvest time, sunlight exposure, post-harvest treatment, extraction methodologies, and experimental conditions, cannot be ruled out [11,30].

In the current research, Zahdi had the second-highest total phenolic content after Kabkab, with a value of 211.1 mg GAE/g DM. This value was remarkably much higher than that found by Molan et al. [31], who reported that Zahdi had a TPC of 4.79 mg GAE/g DM when extracting polyphenols at room temperature using distilled water as a solvent. This significant variation in the obtained results could be explained by differences in the experimental conditions, particularly in the extraction method, where Molan’s study did not perform any stirring, and the matrix was just soaked overnight without multiple extractions [31]. In addition, a study compared the TPC of 14 date seed varieties (including Zahdi and Kabkab), where polyphenols were extracted using 100% water with a solid: solvent ratio of 1:250, with twice repeated extractions at room temperature, and found that Kabakab had the highest TPC (1404 ± 13.52 mg GAE/100 g), followed by Zahdi (1161 ± 104.08 mg GAE/100 g) [32]. This study confirmed our finding that the TPC of Kabkab was higher than that of Zahdi and all other varieties. Furthermore, Majdool, which showed the lowest TPC (63.2 mg GAE/g DM), was previously evaluated and reported to have the lowest TPC value (2088 ± 124 mg GAE/100g) when compared to the other three varieties [33].

### 3.2. Phenolic Profile of Date Seeds

After assessing and comparing the total phenolic content, we found it crucial and interesting to look into the different phenolic compounds present in the six date seed varieties. The results of the HPLC-MS analysis are shown in Table 1. The retention time was used to identify the phenolic compounds, and their concentrations were calculated using the peak areas.

The phenolic compounds in all date seed cultivars varied greatly on average. Caffeic acid appeared to be the dominant phenolic compound with an average of 0.36 mg/g sample, which was followed by proanthocyanidin trimers (0.34 mg/g sample). These were further followed by proanthocyanidin dimers, proanthocyanidin tetramers, and catechin, which all showed significant differences on average as well. Quercetin, on the other hand, was the least abundant phenolic compound. The overall proanthocyanidin dimers content appeared to be 0.13 mg/g on average across the different date seed cultivars. Zahdi had the highest value (0.17 ± 0.02 mg/g), whereas Majdool appeared to have the lowest (0.09 mg/g). In addition, total proanthocyanidin trimers had an average content of 0.34 mg/g. Similar to total proanthocyanidin dimers, Zahdi had the highest concentration (0.41 mg/g), and Majdool had the lowest (0.24 mg/g). The analysis of proanthocyanidin tetramers revealed that Safawi and Zahdi had the highest value of 0.12 mg/g, while Khudari had the lowest (0.07 mg/g). Catechin content ranged from 0.04 mg/g in Majdool to 0.07 mg/g in Zahdi and Kabkab. Moreover, Safawi appeared to have the highest caffeic acid concentration (0.4 mg/g), whereas Khudari had the lowest (0.21 mg/g). Finally, the average Quercetin content was around 0.04 mg/g among all varieties.

The same aforementioned factors that influence the total phenolic content cannot be ruled out here as well. HPLC analysis confirmed the presence of different phenolic compounds, and mainly flavanols seemed to be the dominant family, as it was present in two forms: monomeric (catechin) and polymeric (proanthocyanidin). This is consistent with the findings of earlier investigations [34,35]. Hydroxycinnamic acid (caffeic acid) and flavonols (quercetin) were also detected, with considerably lower concentrations than flavanols. The average caffeic acid concentration (0.36 mg/g) was similar to that previously determined for the Khalas variety (0.34 mg/g). Similarly, the total average of quercetin (0.04 mg/g) was similar to that found by the same previous study with a quercetin value of 0.036 mg/g [12].

### 3.3. Antioxidant Activity of Date Seed Extracts

**Figure 1:**
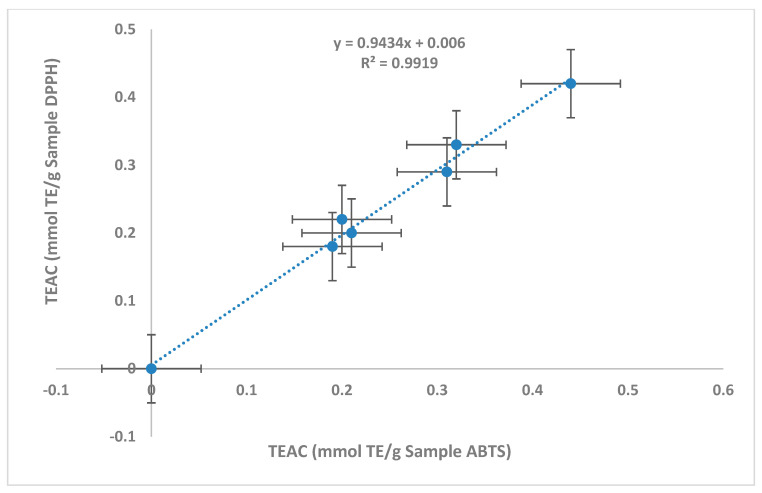
Correlation coefficient (R^2^) between antioxidant assays (ABTS and DPPH) with respect to *TEAC* values (mmol TE/g Sample).

Date seeds were shown to possess powerful antioxidant activities that varied significantly among all varieties. The antioxidant results of the two performed assays (ABTS and DPPH) are shown in Table 2. On average, Trolox equivalent antioxidant capacity (*TEAC*) in ABTS and DPPH appeared to be 0.28 ± 0.03 and 0.27 ± 0.01 mmol TE/g sample, respectively. Those values were not significantly different, indicating a high correlation between both assays. This was further confirmed via linear regression analysis, which revealed a strong correlation (R^2^ > 0.99) (Figure 1). In addition, the average *%DPPH inhibition* was found to be 31.06 ± 1.64%.

In both assays, Kabkab demonstrated the strongest antioxidant effect, with *TEAC* values of 0.44 ± 0.04 and 0.42 ± 0.02 mmol TE/g samples in ABTS and DPPH assays, respectively. Furthermore, the highest value of *%DPPH inhibition* was shown for Kabkab (48.42 ± 2.29%). In contrast, Majdool had the lowest *TEAC* values (0.19 ± 0.01 and 0.18 ± 0.03 mmol TE/g sample in ABTS and DPPH assays, respectively), as well as the lowest % DPPH inhibition (24.82 ± 1.48%) (Table 2). The antioxidant activities of date seeds showed a strong correlation with their TPC values, indicating that the exhibited radical scavenging property is associated with the total phenolic content, and polyphenols are the significant contributors to this effect [36,37,38].

### 3.4. Antibacterial Activity of Date Seed Extracts

#### 3.4.1. Disk Diffusion Assay

The extracts’ antibacterial activities against *S. aureus*, *B. cereus*, *S. typhi*, and *E. coli* were very weak at a 1:100 ratio, where none of the varieties exhibited inhibitory zones against any of the strains after several trials. As a result, other trials were conducted at a different extraction ratio (1:5) for only two date seed varieties (Zahdi and Kabkab) initially, where only Zahdi showed an insignificant inhibition zone of diameter 7 mm against just one bacterial strain (*B. cereus*) (Table S1).

In order to further concentrate the extract and possibly boost its antibacterial effect, a 1:2 solid-to-solvent ratio was used. The order of total phenolic content of the tested date seed varieties varied and was as follows: Sakai had the highest TPC (63.6 mg GAE/g DM), followed by Khudari, Zahdi, Safawi, Kabkab, and lastly Majdool (2.8 mg GAE/g DM) (Table S2). 

The diameters of inhibition zones (mm) of disk diffusion assay are reported in Table 3. Results revealed that the variability in antibacterial activity can be attributed to a variety of differences and the phenolic compounds held in each variety. Similar to antioxidant activity, antibacterial effects demonstrated a high correlation with the total phenolic content. Findings showed the sensitivity of both tested Gram-positive strains (*S. aureus* and *B. cereus*) with the following pattern: Sakai, which appeared to have the highest TPC, was the most powerful, followed by Khudari, Zahdi, and Safawi, all of which had a stronger effect on *B. cereus*. In contrast, no inhibitory zones were observed against Gram-negative bacteria (*E. coli* and *S. typhi*). On the other hand, the low TPC of Kabkab and Majdool (3.2 and 2.8 mg GAE/g DM) seemed insufficient to show inhibitory zones against any of the examined bacterial strains. Additionally, this was the case in Safawi, which showed some effect against *B. cereus* but none against all other strains (Figures S1–S3). Our results confirm that polyphenols are the major contributors to the observed antibacterial activity.

The cutoffs reported by the Clinical & Laboratory Standards Institute (CLSI) classify antibacterial activity based on the zone of inhibition diameters as follows: less than 10 mm is considered weak, between 10 and 13 mm is regarded as moderate activity, and greater than 13 mm is deemed high activity [38]. For *S. aureus*, the highest zone of inhibition was shown by the Sakai Saudi variety (11 mm), which is considered a moderate activity. This result was consistent with that of a study conducted to investigate the antibacterial ability of Mosaifah Saudi aqueous extract against the same bacterial strain, which showed a 10 mm diameter zone of inhibition. However, Mosaifah demonstrated an inhibitory zone of 9 mm against *E. coli* [39]. Another study assessed the antibacterial potentials of aqueous Khudari seed extract, which exhibited no effect against *S. aureus* (ATCC29213) [40]. To our knowledge, so far, no studies have evaluated the antibacterial activity of date seeds against *S. typhi*. This is also the first study to assess the effect of aqueous date seed extracts against *B. cereus*.

It is important to note that the difference in antibacterial activity is highly dependent on the tested bacterial strain, with a stronger effect against Gram-positive bacterial strains (*S. aureus* and *B. cereus*) than Gram-negative bacterial strains (*S. typhi* and *E. coli*). In addition, the effect on *B. cereus* appeared to be stronger than on *S. aureus*. Our study findings demonstrate the complexity of compounds involved in antibacterial activity, even among varieties from the same origin. The ATCC numbers of the bacterial strains used were not included in most studies. Hence, differences in the obtained results against the same bacterial strains due to different ATCC numbers are possible. Moreover, the extraction solvent and methodology can both play a major role [39].

#### 3.4.2. Minimum Inhibitory Concentration (MIC)

The two varieties that exerted the highest antibacterial efficacy in the disk diffusion method (Sakai and Khudari) were chosen to determine the MIC against all the bacterial strains. Table 4 shows the MIC determination of Sakai against Gram-positive bacteria (*S. aureus* and *B. cereus*). Figures for all MIC determination experiments can be found in Figures S4–S7.

The obtained results confirm the findings of the disk diffusion experiment. *S. aureus* and *B. cereus* appeared to be inhibited by date seed extracts. The MIC for both Khudari and Sakai was determined to be 41.6 mg/mL against *S. aureus*, whereas it was lower against *B. cereus* (20.8 mg/mL), indicating a stronger effect. In contrast, both Gram-negative bacterial strains (*S. typhi* and *E. coli*) were not inhibited by both varieties, where visible turbidity was seen in all the tubes even at higher concentrations of date seed extracts. In a prior investigation, the MIC of Kabkab and Zahdi against *S. aureus* was reported as 1.56 mg/mL, which is considerably lower than our findings. In line with our results, the study reported no antibacterial activity against *E. coli* (ATCC 25922) [41].

Our findings demonstrate that antibacterial activity is generally dependent on the target bacterium and is selective against specific bacterial strains. The variations in susceptibilities of bacteria can be correlated with differences in the cell wall structures of the bacterial strain itself. In fact, the antibacterial strength of the phenolic compounds is associated with their ability to cross the outer layer of Gram-positive bacteria and interact with its peptidoglycan layer, hence weakening cell integrity and leading to an inhibitory and/or bactericidal effect, depending on the bacterial strain’s sensitivity to osmotic pressure and ionic strength. As a defense mechanism, the outer membrane of Gram-negative bacteria includes a lipopolysaccharide layer, which acts as a strong barrier that obstructs the interaction of polyphenols with the peptidoglycan layer [42].

### 3.5. Anti-tumor Activity of Date Seeds

**Figure 2:**
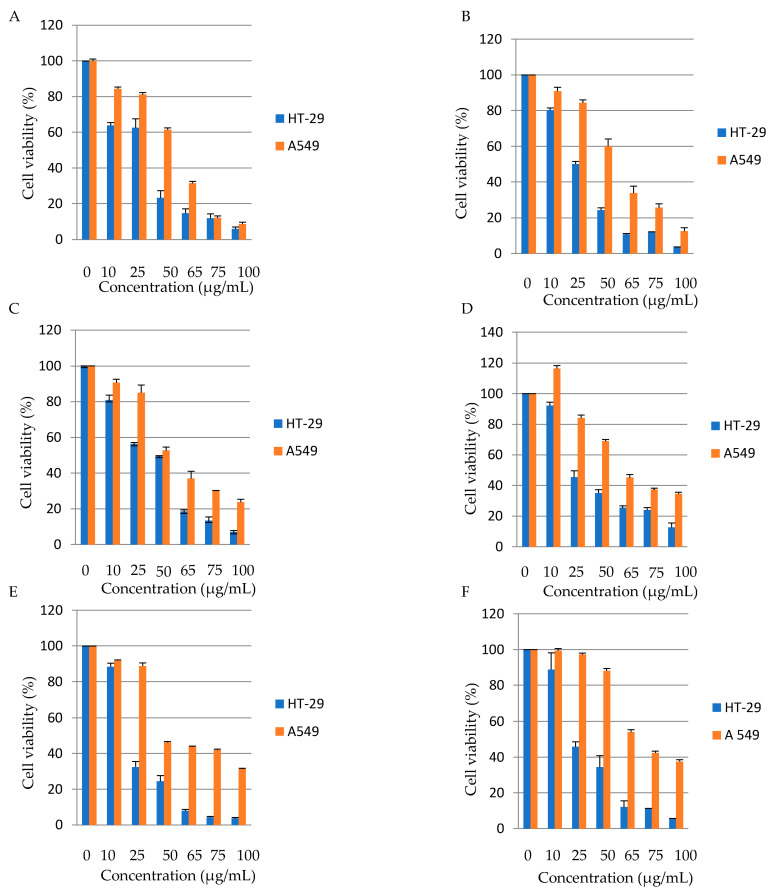
The anti-tumor activity of date seeds on HT-29 and A549 cells at different concentrations. (**A**) Khudari, (**B**) Sakai, (**C**) Safawi, (**D**) Majdool, (**E**) Zahdi, and (**F**) Kabkab. Represented data are the average of 3 different determinations ± SD.

All date seed varieties showed dose-dependent anti-tumor activity on both cells HT-29 and A549 at different concentrations and times. Khudari date seed variety was effective in reducing cell viability by 36.1% in HT-29 cells at 10 µg/mL and by 38.51% at 50 µg/mL in A549 cells, with respective IC50 of 36.65 µg/mL and 57.8 µg/mL. Sakai date was effective in lowering cell viability by 49.84% in HT-29 cells at 25 µg/mL and by 39.8% at 50 µg/mL in A549 cells, with respective IC50 of 29.86 µg/mL and 55.05 µg/mL. As for Safawi, it had the ability to decrease cell viability by 43.76% in HT-29 cells at 25 µg/mL and by 47.4% at 50 µg/mL in A549 cells, with respective IC50 of 46.85 µg/mL and 48.3 µg/mL. Furthermore, the Majdool variety had the ability to reduce cell viability by 54.6% in HT-29 cells at 25 µg/mL and by 31% at 50 µg/mL in A549 cells, with respective IC50 of 29.68 µg/mL and 70.13 µg/mL. Zahdi was effective in decreasing cell viability by 67.5% in HT-29 cells at 25 µg/mL and by 53.7% at 50 µg/mL in A549 cells, with respective IC50 of 18.9 µg/mL and 54 µg/mL. Furthermore, Kabkab date seed extract was efficient in reducing cell viability by 54.16% in HT-29 cells at 25 µg/mL and by 45.96% but at 65 µg/mL in A549 cells, with respective IC50 of 28.64 µg/mL and 59 µg/mL. All date seed varieties showed higher effectiveness in reducing cell viability in HT-29 than in A549 with the most effective variety being Khudari (Figure 2) (Table 5). The IC_50_ values for all varieties were higher in the lung cancer cell line, indicating that date seed aqueous extracts were more effective against colon cancer cells than lung cancer cells. It is worth noting that in contrast to other studies, our study reached a maximum dosage of 100 µg/mL, which almost completely reduced HT-29 and A549 viability [43]. No previous research evaluated the anti-tumor activity of date seeds against HT-29 and A549 specifically. Previous studies found that date seeds had dose-dependent cytotoxic effects against various cancer cell lines, including HepG-2 (liver cancer), Caco-2 (colon cancer), MDA (breast cancer), and PC3 (prostate cancer) cell lines only at high concentrations starting at 1000 µg/mL after 24 and 48 h of treatment [13,44]. The diversity in the obtained results can be explained by differences in cultivars and their phenolic profile, extraction solvent, susceptibility of cancer cells, concentrations of date seed extracts, and duration of incubation.

Our study included the examination of the anti-tumor activity of date seeds against the HT-29 cell line, i.e., the in vitro nutritional tests on a model of the digestive system [45]. In fact, colorectal cancer is the third most frequently diagnosed type of cancer after lung and breast cancer and the second cancer killer worldwide. The current treatment mainly relies on chemotherapy, which can affect cell cycles of tumors as well as non-malignant cells and produce adverse side effects such as nausea, vomiting, diarrhea, alopecia (hair loss), myelosuppression, depression, and many others [46]. For this reason, the current approach in this field is to introduce natural bioactive compounds, mainly polyphenols, and investigate their potential anti-tumor properties against cancer cells for possible replacement of existing chemotherapeutic medicines with the goal of reducing patient suffering. Our study intended the use of distilled water as a solvent for extracting polyphenols from date seeds so that its potential consumption and passage through the colon would improve gastrointestinal function and reduce the viability of cancer cells if they were existent.

Overall, the antioxidant and antibacterial activities appeared to be correlated with the TPC of date seeds. Furthermore, the antioxidant activity seemed to be correlated with catechin, where Kabkab, which had the highest antioxidant potential, appeared to have the highest value of catechin (0.07 mg/g), while Majdool possessing the least radical scavenging capacity, had the lowest catechin value (0.04 mg/g). Previous studies have revealed the biological effectiveness of catechin in scavenging reactive oxygen species owing to their hydroxyl-rich structure [47,48,49,50]. However, this is not the case in anti-tumor activity. In addition, this activity could not be attributed to any of the detected phenolic compounds, which suggests the role of a certain polyphenol that was not detected on HPLC analysis due to limited standards used. P-coumaric acid, sinapic acid, and chlorogenic acid were found to be dominant in date seeds but were not included as standards in our HPLC analysis for comparison between the examined date seed varieties [12,37,51,52,53].

### 3.6. Date Seed Oil Yield and Fatty Acids Content

Table 6 shows the yield and fatty acids composition of the six extracted date seed oils. The oil content and yield varied significantly among date seed varieties and appeared to be 0.36 ± 0.03 g and 3.81 ± 0.26% on average. The maximum amount of fats and oil yield was shown in the Sakai variety (0.42 ± 0.04 g and 4.89 ± 0.11%). In contrast, the Zahdi variety had the lowest amount of fat and oil yield (0.26 ± 0.00 g and 2.59 ± 0.03%).

The two most dominating fatty acids appeared to be oleic acid and lauric acid, with an average concentration of 0.1 g/100 g. This was followed by mystric, palmitic, and stearic acids, with values of 0.07, 0.04, and 0.03 g/100 g, respectively, which all showed significant differences. Capric acid, on the other hand, was the least abundant fatty acid, with a negligible value of 0.005 g/100 g.

Our findings were consistent with those of Mrabet et al., who reported that date seed oil appeared to be composed of both saturated and unsaturated fatty acids, with oleic acid and lauric acid predominating [54]. Oleic acid is a highly valued monounsaturated fatty acid due to its excellent stability and auspicious health benefits. It is regarded as a crucial fatty acid due to its low saturation level, its high radical scavenging properties, as well as its ability to prevent atherosclerosis and heart disease by lowering high blood cholesterol levels. Furthermore, it is widely known that oil rich in unsaturated fatty acids might lower the risk of cardiovascular disease and inflammatory conditions [55]. On the other hand, lauric acid, which is classified as a saturated fatty acid, was found to be preferable to trans-fatty acids in terms of disruption of lipid profile [56]. It also appeared to possess some antibacterial properties, as it inhibited the growth of pathogenic bacteria (especially *S. aureus*) and reduced their ability to produce toxins [37,49,50]. Interestingly, lauric and mystric acids, which are both present in date seed oils, revealed preventive effects on the development of prostatic hyperplasia [57,58].

## 4. Conclusions

The present work concluded that date seeds are a great source of bioactive compounds, mainly polyphenols, which appeared to vary depending on the variety and origin. Kabkab (Iran) had the highest TPC value, whereas Majdool (Jordan) had the lowest. Flavanols appeared to be the dominating family of phenolic compounds. Date seeds’ promising antioxidant activity were correlated with TPC in all varieties, suggesting that polyphenols, especially catechin, are key contributors to this power. Khudari and Sakai date seeds showed the highest antibacterial activities against Gram-positive strains, especially *B. cereus*. In contrast, Gram-negative bacterial strains appeared to be resistant to all varieties. Sakai and Khudari demonstrated the strongest anticancer activity, with a stronger effect on the colon than on lung cancer cells. Date seed oil was found to be rich in oleic and lauric acids.

The findings of this research suggest the usefulness of date seeds by food manufacturers as functional food additives and nutraceuticals. The use of distilled water was intended to mimic the way people might incorporate date seeds into their diets. However, extensive preclinical and clinical studies are needed to assess the safety of date seeds on humans. As no single variety consistently appeared to be most powerful in the assessed biological activities, future in-depth research about the synergistic activity of different varieties and the underlying mechanisms of date seed polyphenols need to be conducted.

## Figures and Tables

**Table 1:** Phenolic compounds (mg/g) and total phenolic content (TPC) (mg GAE/g DM) of the six date seed varieties.

Phenolic Compounds	Retention Time (Minutes)	Date Seed Variety						
		Khudari	Sakai	Safawi	Majdool	Zahdi	Kabkab	Average
Proanthocyanidin dimer 1	6.85	0.07 ± 0.00	0.07 ± 0.00	0.02 ± 0.00	0.02 ± 0.00	0.11 ± 0.02	0.08 ± 0.00	0.06 ± 0.00
Proanthocyanidin dimer 2	7.17	0.05 ± 0.00	0.07 ± 0.00	0.11 ± 0.00	0.07 ± 0.00	0.06 ± 0.00	0.05 ± 0.00	0.06 ± 0.00
Total Proanthocyanidin dimers	_	0.12 ± 0.00 ^e^	0.14 ± 0.00 ^b^	0.13 ± 0.00 ^cd^	0.09 ± 0.00 ^f^	0.17 ± 0.02 ^a^	0.13 ± 0.00^c^	0.13 ± 0.00 ^c^
Proanthocyanidin trimer 1	7.73	0.11 ± 0.00	0.1 ± 0.00	0.1 ± 0.00	0.07 ± 0.00	0.09 ± 0.00	0.07 ± 0.00	0.09 ± 0.00
Proanthocyanidin trimer 2	8.08	0.07 ± 0.00	0.11 ± 0.00	0.09 ± 0.00	0.06 ± 0.00	0.11 ± 0.00	0.11 ± 0.01	0.09 ± 0.00
Proanthocyanidin trimer 3	8.77	0.09 ± 0.00	0.10 ± 0.00	0.09 ± 0.00	0.06 ± 0.00	0.10 ± 0.00	0.09 ± 0.01	0.08 ± 0.00
Proanthocyanidin trimer 4	10.71	0.03 ± 0.00	0.03 ± 0.00	0.06 ± 0.00	0.03 ± 0.00	0.07 ± 0.00	0.05 ± 0.00	0.04 ± 0.00
Proanthocyanidin trimer 5	10.98	0.03 ± 0.00	0.03 ± 0.00	0.04 ± 0.00	0.02 ± 0.00	0.04 ± 0.00	0.03 ± 0.00	0.03 ± 0.00
Total Proanthocyanidin trimers	_	0.33 ± 0.00 ^e^	0.37 ± 0.01 ^b^	0.38 ± 0.00 ^bc^	0.24 ± 0.00 ^f^	0.41 ± 0.00 ^a^	0.35 ± 0.02 ^d^	0.34 ± 0.00 ^b^
Proanthocyanidin tetramer 1	11.89	0.02 ± 0.00	0.03 ± 0.00	0.04 ± 0.00	0.03 ± 0.00	0.05 ± 0.00	0.03 ± 0.00	0.03 ± 0.00
Proanthocyanidin tetramer 2	12.61	0.03 ± 0.01	0.03 ± 0.00	0.04 ± 0.00	0.03 ± 0.00	0.04 ± 0.00	0.03 ± 0.00	0.03 ± 0.00
Proanthocyanidin tetramer 3	14.05	0.02 ± 0.00	0.03 ± 0.00	0.04 ± 0.00	0.03 ± 0.00	0.03 ± 0.00	0.03 ± 0.00	0.03 ± 0.00
Total Proanthocyanidin tetramers	_	0.08 ± 0.01 ^ef^	0.08 ± 0.00 ^e^	0.12 ± 0.00 ^a^	0.09 ± 0.01 ^c^	0.12 ± 0.00 ^ab^	0.09 ± 0.00 ^cd^	0.09 ± 0.00 ^d^
Catechin	7.47	0.05 ± 0.00 ^e^	0.06 ± 0.00 ^c^	0.06 ± 0.00 ^cd^	0.04 ± 0.00 ^f^	0.07 ± 0.00 ^a^	0.07 ± 0.00 ^ab^	0.06 ± 0.00 ^e^
Caffeic acid derivative 1	8.93	0.11 ± 0.00	0.12 ± 0.00	0.13 ± 0.00	0.12 ± 0.00	0.12 ± 0.00	0.11 ± 0.00	0.12 ± 0.00
Caffeic acid derivative 2	9.10	0.1 ± 0.00	0.11 ± 0.00	0.14 ± 0.00	0.14 ± 0.00	0.13 ± 0.00	0.12 ± 0.00	0.12 ± 0.00
Caffeic acid derivative 3	9.49	ND	0.09 ± 0.00	0.12 ± 0.00	0.13 ± 0.00	0.12 ± 0.00	ND	0.11 ± 0.00
Caffeic acid derivative 4	9.95	ND	ND	0.01 ± 0.00	ND	ND	ND	0.01 ± 0.00
Caffeic acid derivative 5	11.30	ND	ND	ND	ND	ND	ND	ND
Caffeic acid derivative 6	11.60	ND	ND	ND	ND	ND	ND	ND
Total caffeic acids	_	0.21 ± 0.00 ^f^	0.32 ± 0.00 ^d^	0.4 ± 0.00 ^a^	0.39 ± 0.00 ^b^	0.37 ± 0.00 ^c^	0.23 ± 0.00 ^e^	0.36 ± 0.00 ^a^
Quercetin	15.50	0.03 ± 0.00 ^bc^	0.05 ± 0.00 ^a^	ND	0.03 ± 0.00 ^bcd^	ND	0.03 ± 0.00 ^b^	0.04 ± 0.00 ^f^
Total Phenolic Content	_	77.1 ± 0.3 ^e^	203.2 ± 2.3 ^c^	90.6 ± 1.3 ^d^	63.2 ± 0.1 ^f^	211.1 ± 2.3 ^b^	211.1 ± 2.3 ^b^	154.2 ± 1.3

Results are reported as mean ± standard deviation. Values with different alphabetical letters appeared significantly different (*p* < 0.05). ND: Not determined.

**Table 2:** Antioxidant activity of the six date seed varieties assessed by two methods (ABTS and DPPH). Results are expressed as Trolox equivalent antioxidant capacity (*TEAC*) (mmol TE/g) and *%DPPH inhibition*.

Date Seed Variety	ABTS	DPPH	
	*TEAC*_ABTS_	*TEAC*_DPPH_	*%DPPH Inhibition*
Khudari	0.21 ± 0.01 ^d^	0.20 ± 0.00 ^e^	22.16 ± 0.11 ^e^
Sakai	0.31 ± 0.00 ^c^	0.29 ± 0.01 ^c^	33.68 ± 1.53 ^c^
Safawi	0.20 ± 0.01 ^de^	0.22 ± 0.01 ^d^	24.72 ± 1.53 ^d^
Majdool	0.19 ± 0.01 ^f^	0.18 ± 0.03 ^f^	19.76 ± 3.38 ^f^
Zahdi	0.32 ± 0.07 ^b^	0.33 ± 0.01 ^b^	37.66 ± 0.98 ^b^
Kabkab	0.44 ± 0.04 ^a^	0.42 ± 0.02 ^a^	48.42 ± 2.3 ^a^
Average	0.28 ± 0.02	0.27 ± 0.01	31.06 ± 1.64

Results are reported as mean ± standard deviation. Values with different alphabetical letters appeared significantly different (*p* < 0.05).

**Table 3:** Antibacterial activities (disk diffusion) of the six date seed extracts against four bacterial strains.

Date Seed Variety	Diameter of Inhibition Zone (mm)			
	*Staphylococcus aureus*	*Bacillus cereus*	*Salmonella typhi*	*Escherichia* *coli*
Khudari	9 ± 0.35	12 ± 0.56	NI	NI
Sakai	11 ± 0.75	13 ± 0.94	NI	NI
Safawi	NI	8.5 ± 1.03	NI	NI
Majdool	NI	NI	NI	NI
Zahdi	8 ± 0.87	9 ± 0.43	NI	NI
Kabkab	NI	NI	NI	NI
Gentamycin (50 μg)	31 ± 1.47	29 ± 0.59	30 ± 1.05	30 ± 0.95

Data are means of three replicates (n = 3) ± standard deviation. NI: no inhibition.

**Table 4:** Minimum inhibitory concentration (MIC) (mg/mL) of Khudari and Sakai date seed extracts against four bacterial strains.

Date Seed Variety	Minimum Inhibitory Concentration (MIC) (mg/mL)			
	*Staphylococcus aureus*	*Bacillus cereus*	*Salmonella typhi*	*Escherichia* *coli*
Khudari	41.6	20.8	NI	NI
Sakai	41.6	20.8	NI	NI

NI: no inhibition.

**Table 5:** IC50 for all date seed varieties on HT-29 and A549 cells (µg/mL).

Date Seed Variety	HT-29	A549
Khudari	36.65 ± 1.38	57.8 ± 1.02
Sakai	29.86 ± 1.17	55.05 ± 1.02
Safawi	46.85 ± 3.63	48.3 ± 1.01
Majdool	29.68 ± 2.04	70.13 ± 4.70
Zahdi	18.9 ± 2.63	54 ± 1.17
Kabkab	28.64 ± 1.58	59 ± 1.01

**Table 6:** The amount of obtained oil (g), yield (%), and fatty acids (g/100 g) detected in the six date seed oils.

Date Seed Variety	Amount of Fat (g)	Oil Yield (%)	Fatty Acids (g/100 g)						
			Capric Acid	Lauric Acid	Mystric Acid	Palmitic Acid	Stearic Acid	Oleic Acid	Average
Khudari	0.37 ± 0.06 ^c^	3.79 ± 0.42 ^c^	0.004 ± 0.01	0.07 ± 0.01 ^f^	0.08 ± 0.01 ^b^	0.05 ± 0.01 ^a^	0.05 ± 0.01 ^a^	0.12 ± 0.02 ^c^	0.37 ± 0.06 ^b^
Sakai	0.42 ± 0.04 ^a^	4.89 ± 0.11 ^a^	0.005 ± 0.00	0.1 ± 0.01 ^bc^	0.08 ± 0.01 ^a^	0.05 ± 0.01 ^ab^	0.04 ± 0.00 ^b^	0.13 ± 0.01 ^a^	0.4 ± 0.04 ^a^
Safawi	0.36 ± 0.03 ^cde^	3.6 ± 0.29 ^e^	0.005 ± 0.00	0.11 ± 0.01 ^b^	0.07 ± 0.01 ^cd^	0.04 ± 0.00 ^cd^	0.03 ± 0.00 ^cd^	0.11 ± 0.01 ^d^	0.36 ± 0.03 ^cd^
Majdool	0.38 ± 0.01 ^b^	4.32 ± 0.26 ^b^	0.007 ± 0.00	0.12 ± 0.00 ^a^	0.07 ± 0.00 ^bc^	0.04 ± 0.00 ^c^	0.03 ± 0.00 ^cde^	0.1 ± 0.00 ^de^	0.37 ± 0.00 ^c^
Zahdi	0.26 ± 0.00 ^f^	2.59 ± 0.03 ^f^	0.005 ± 0.00	0.08 ± 0.00 ^e^	0.05 ± 0.00 ^f^	0.03 ± 0.00 ^f^	0.02 ± 0.00 ^f^	0.08 ± 0.00 ^f^	0.27 ± 0.00 ^f^
Kabkab	0.36 ± 0.06 ^cd^	3.65 ± 0.59 ^d^	0.005 ± 0.00	0.1 ± 0.02 ^d^	0.06 ± 0.01 ^de^	0.04 ± 0.01 ^cde^	0.03 ± 0.01 ^c^	0.12 ± 0.02 ^b^	0.35 ± 0.00 ^f^
Average	0.36 ± 0.03	3.81 ± 0.26	0.005 ± 0.00 ^f^	0.1 ± 0.01 ^ab^	0.07 ± 0.01 ^c^	0.04 ± 0.00 ^d^	0.03 ± 0.00 ^de^	0.11 ± 0.01 ^a^	_

Mean ± SD. Values with different alphabetical letters appeared significantly different (*p* < 0.05). Values with no letters indicate no significant difference.

## Data Availability

Data are contained within the article and supplementary materials.

## Supplementary Materials

The following supporting information can be downloaded at https://www.mdpi.com/article/10.3390/foods13010126/s1. Table S1: Antibacterial activity (disk diffusion trial) of Zahdi and Kabkab date seeds extracts against the four tested bacterial strains at 1:5 extraction ratio. Table S2: Total phenolic content (TPC) of date seeds extracts (mg GAE/g DM). Figure S1: Antibacterial activity (disk diffusion) of the six date seeds extracts. Figure S2: Antibacterial activity of Sakai against Staphylococcus aureus showing the controls used; Figure S3: Antibacterial activity (disk diffusion) of the six date seeds extracts; Figure S4: MIC determination of Khudari extract against gram-positive bacterial strains; Figure S5: MIC determination of Khudari against gram-negative bacterial strains; Figure S6: MIC determination of Sakai extract against gram-positive bacterial strains; Figure S7: MIC determination of Sakai against gram-negative bacterial strains.
