# Supplementary material for: Evaluation of the Phenolic Composition and Biological Activities of Six Aqueous Date (Phoenix dactylifera L.) Seed Extracts Originating from Different Countries: A Comparative Analysis
Source: Foods. 2023 Dec 29;13(1):126. doi: 10.3390/foods13010126 (PMC10778786; doi:10.3390/foods13010126)
Supplement: Supplementary file 1 [file foods-13-00126-s001.zip › foods-2780466-supplementary.pdf]

Table S1. Antibacterial activity (disk diffusion trial) of Zahdi and Kabkab date seeds extracts against the four tested bacterial strains at 1:5 extraction ratio.

**Date Seeds Variety**	**Diameter of inhibition zone (mm)**			
	Staphylococcus aureus	***Bacillus cereus***	***Salmonella typhi***	***Escherichia coli***
**Zahdi**	NI	7	NI	NI
**Kabkab**	NI	NI	NI	NI

Table S2. Total phenolic content (TPC) of date seeds extracts (mg GAE/g DM) at 1:2 extraction ratio.

**Date Seeds Variety**	**TPC (mg GAE/g DM)**
**Khudari**	14.1 ±1.2^{b}
**Sakai**	63.6 ±1.5^{a}
**Safawi**	4.1 ±0.9^{d}
**Majdool**	2.8 ±1.3^{f}
**Zahdi**	5.7 ±0.7^{c}
**Kabkab**	3.2 ±0.9^{e}
**Average**	15.6 ±1.1

Mean ± standard deviation. Values with different alphabetical letters appeared significantly different (P <0.05).

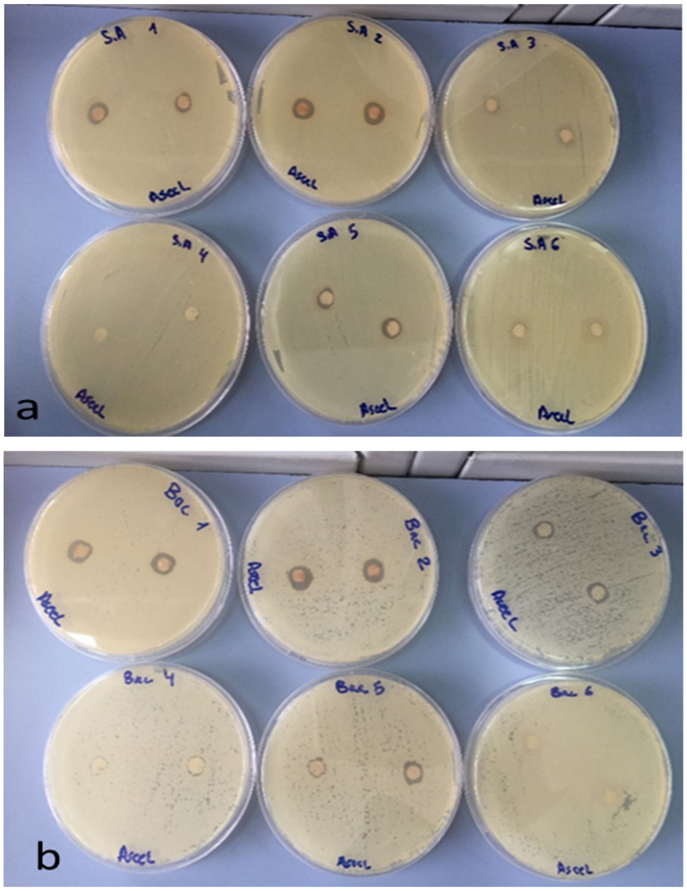

Figure S1. Antibacterial activity (disk diffusion) of the six date seeds extracts. (1) Khudari, (2) Sakai, (3) Safawi, (4) Majdool, (5) Zahdi, (6) Kabkab against gram-positive bacteria: (a) S.A: *Staphylococcus aureus* and (b) Bac: *Bacillus cereus*.

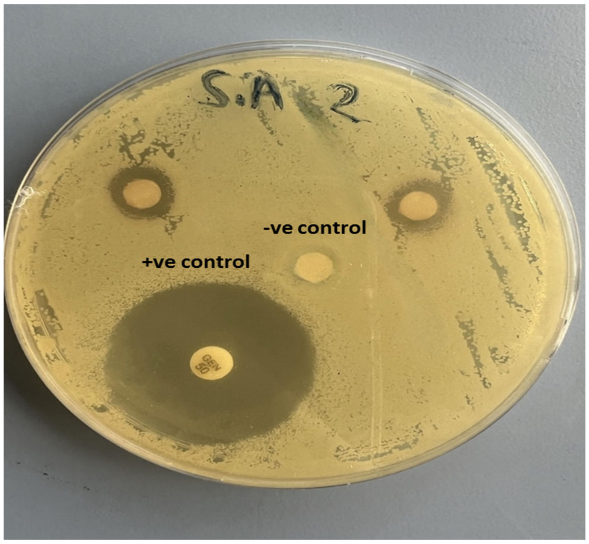

Figure S2. Antibacterial activity of Sakai against *Staphylococcus aureus* showing the controls used:

(-ve) negative control (sterile distilled water), and (+ve) positive control (Gentamycin 50 μg).

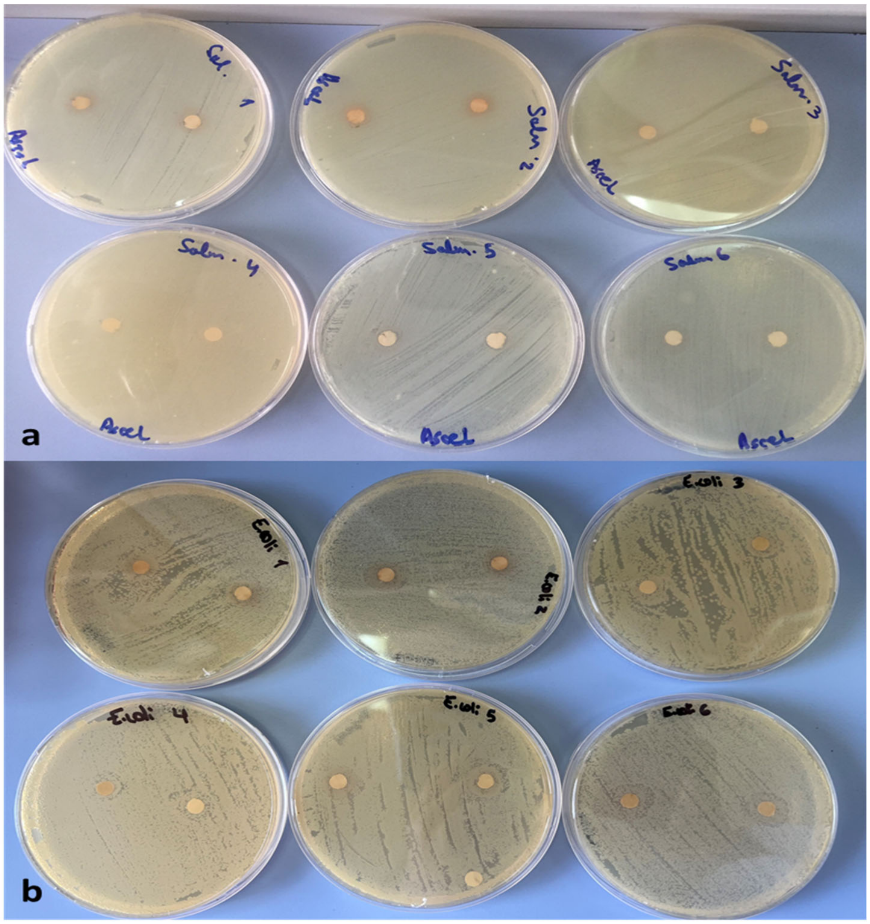

Figure S3. Antibacterial activity (disk diffusion) of the six date seeds extracts

(**1**) Khudari, (**2**) Sakai, (**3**) Safawi, (**4**) Majdool, (**5**) Zahdi, (**6**) Kabkab against: (**a**) *Salmonella* Typhi and (**b**) *Escherichia coli*

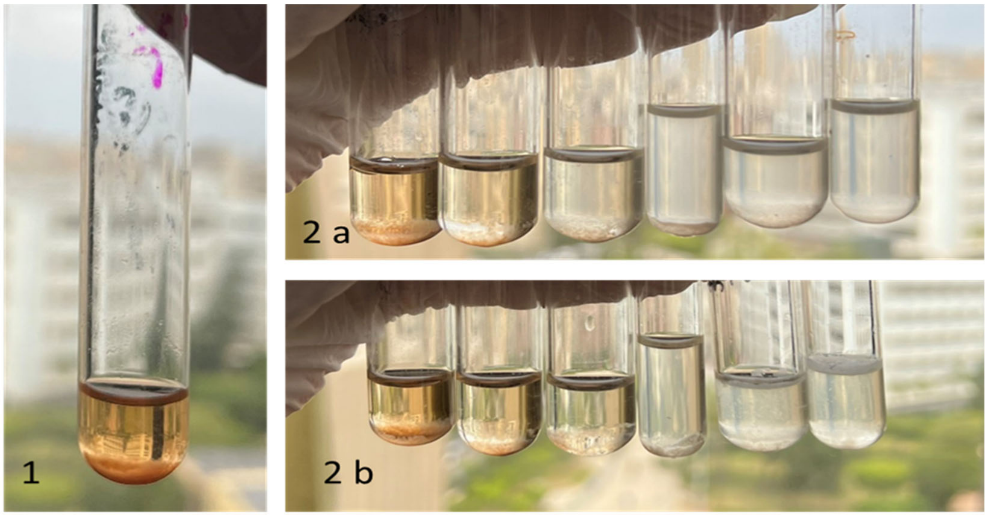

Figure S4. MIC determination of Khudari extract against gram-positive bacterial strains

(**1**) Control (broth + Khudari extract)

(**2**) Broth dilution: (**a**) *Staphylococcus aureus*, (**b**) *Bacillus cereus*

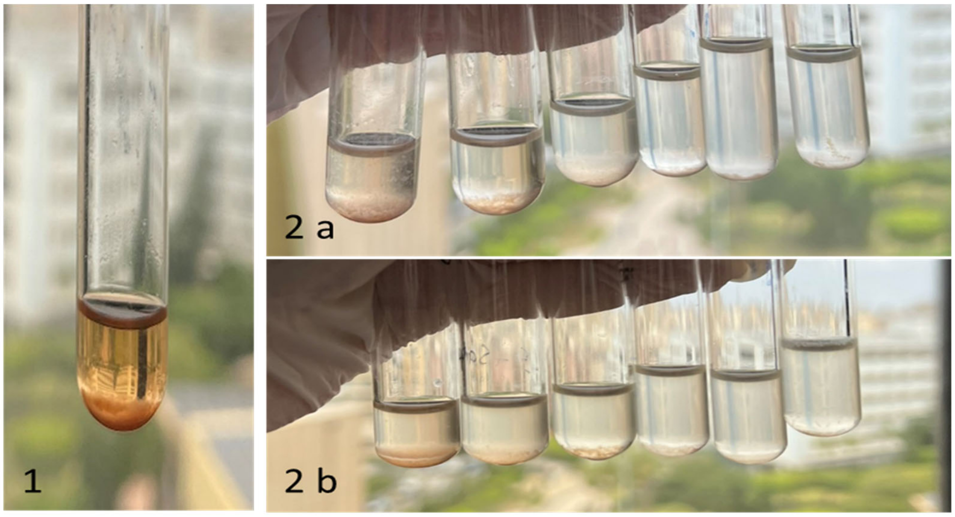

Figure S5. MIC determination of Khudari against gram-negative bacterial strains

(1) Control (broth + Khudari extract)

(2) Broth dilution: (a) *Salmonella* Typhi and (b) *Escherichia coli*

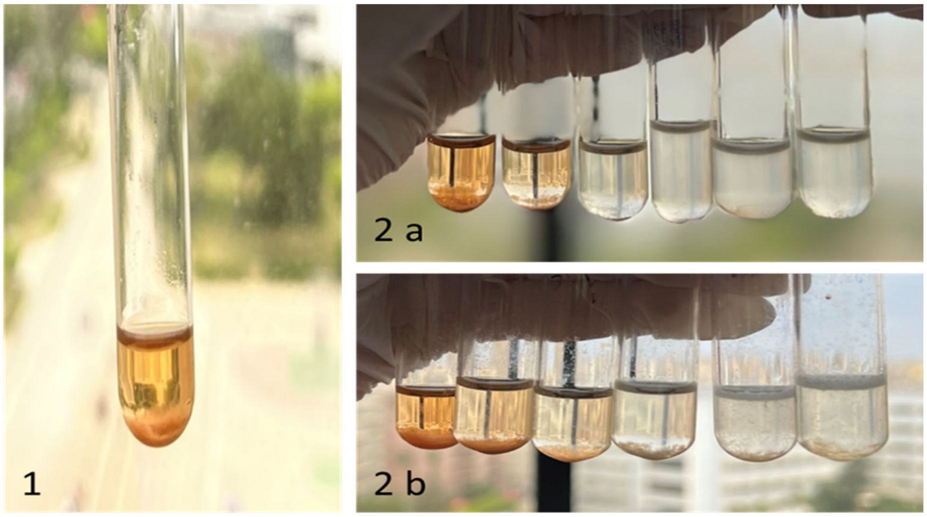

Figure S6. MIC determination of Sakai extract against gram-positive bacterial strains

(**1**) Control (broth + Sakai extract)

(**2**) Broth dilution: (**a**) *Staphylococcus aureus*, (**b**) *Bacillus cereus*

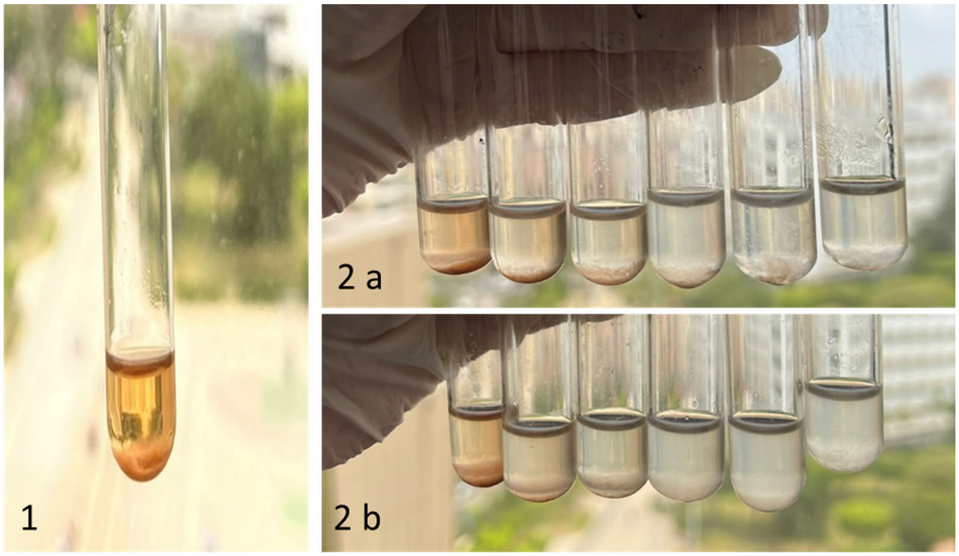

Figure S7. MIC determination of Sakai against gram-negative bacterial strains

(1) Control (broth + Sakai extract)

(2) Broth dilution: (a) *Salmonella* Typhi and (b) *Escherichia coli*
